# Antiviral effects against EV71 of pimprinine and its derivatives isolated from Streptomyces sp

**DOI:** 10.1186/s12985-014-0195-y

**Published:** 2014-11-20

**Authors:** Yanhong Wei, Wei Fang, Zhongyi Wan, Kaimei Wang, Qingyu Yang, Xiaofeng Cai, Liqiao Shi, Ziwen Yang

**Affiliations:** College of Life Sciences, Wuhan University, Wuhan, 430072 P. R. of China; National Biopesticide Engineering Research Center, Hubei Academy of Agricultural Sciences, Wuhan, 430064 P. R. of China; Kekulé-Institute of Organic Chemistry and Biochemistry, University of Bonn, Bonn, 53121 German

**Keywords:** Pimprinine family of compounds, Enteroviruses 71, Broad-spectrum antiviral activity, Mode of action

## Abstract

**Background:**

The pimprinine family of compounds represent very important and promising microbial metabolites for drug discovery. However, their ability in inhibiting viral infections has not yet been tested.

**Methods:**

The antiviral activity of the pimprinine family of compounds was evaluated by determining the cytopathic effect (CPE), cell viability or plaque-forming unit (PFU), and virus yield. The mechanism of action against EV71 was determined from the virucidal activity, and effective stage and time-of-addition assays. The effects on EV71 replication were evaluated further by determining viral RNA synthesis, protein expression and cells apoptosis using the SYBR Green assays, immunofluorescence assays and flow cytometric assays, respectively.

**Results:**

Pimprinethine, WS-30581 A and WS-30581 B inhibited EV71-induced CPE, reduced progeny EV71 yields, as well as prevented EV71-induced apoptosis in human rhabdomyosarcoma (RD) cells. These compounds were found to target the early stages of the EV71 replication in cells including viral RNA replication and protein synthesis. They also showed antiviral activity against ADV-7, and were slightly active against CVB3, HSV-1 and H1N1 with a few exceptions. Pimprinine was slightly active or inactive against all the viruses tested. The mechanisms by which these compounds act against the viruses tested may be similar to that demonstrated for EV71.

**Conclusion:**

The data described herein demonstrate that the pimprinine family of compounds are inhibitors effective against the replication of EV71 and ADV-7, so they might be feasible therapeutic agents for the treatment of viral infections.

## Background

Enterovirus 71 (EV71) is a single positive-stranded RNA virus that belongs to the *Enterovirus* genus of the Picornaviridae family. It was first isolated and characterized from cases of neurological disease in the United States in 1969 [1], subsequent outbreaks of EV71 infections have been reported around the world especially in the Asia-Pacific region [2-7], which mainly affected young children. Clinical manifestations have ranged from mild hand-foot-mouse disease (HFMD) to severe encephalitis and pulmonary edema and even death [8,9]. According to reports from the Chinese Center for Disease Control and Prevention (CCDC), HFMD was listed as the most common category-C infectious disease from 2009 to 2011, based on incidence and death rate, with more than 500 deaths in over 1,600,000 cases of EV71 infection reported in China in 2011 alone [9]. There is currently no vaccine or specific medication for EV71 infections [9], highlighting the urgency and significance of developing suitable anti-EV71 agents. Hence, greater effort needs to be put into developing drugs to conquer the EV71 infections.

Coxsackievirus B3 (CVB3) [10], adenovirus 7 (ADV-7) [11], herpes simplex virus 1 (HSV-1) [12] and influenza virus (H1N1) [13] infections cause common diseases in humans. However, there exists no specific drug that has been approved for the treatment of CVB3 and ADV-7 infections [11]. Also, drug-resistant viral strains and several side effects of drugs used to treat HSV-1 and H1N1, have become more prevalent [14,15]. These emerging problems highlight the need for new, effective and well-tolerated antiviral drugs.

Indole alkaloids have received significant attention during the past decade due to their diverse biological activities. Members of the pimprinine (5, 30-indolyl-2-methyloxazole) family, pimprinethine (pimprinine (n-ethyl) homologue), WS-30581 A and WS-30581 B (pimprinine (n-propyl and n-butyl) derivatives), as natural indole alkaloids, have been isolated from various microbial fermentation broths and have been demonstrated to exhibit broad pharmaceutical activities [16-18]. Pimprinine is an effective inhibitor of monoamine oxidase (MAO) and has been reported to have promising anticonvulsant and antitremorine activity [19]; WS-30581 A and WS-30581 B exhibit significant inhibitory effects on platelet aggregation and have anti-thrombolytic activity in *vitro* [18]. Nitrogen- and oxygen- containing five-membered heterocyclic compounds have been reported to be structures that play key roles in the activities of many biologically interesting natural products and useful therapeutic agents [20]. Therefore, the pimprinine family of compounds may represent a group of very important and promising microbial metabolites in the search for novel drugs. However, the ability of these compounds to inhibit viral infections has not yet been tested. Herein, we report that pimprinine, pimprinethine, WS-30581 A and WS-30581 B are inhibitors of EV71 infection *in vitro*, and we have identified their preliminary modes of action. Moreover, the antiviral activities of the pimprinine family of compounds against CVB3, ADV-7, HSV-1 and H1N1 infections were also described for the first time.

## Results

### The antiviral activity of pimprinine, pimprinethine, WS-30581 A and WS-30581 B against EV71

**Figure 1:**
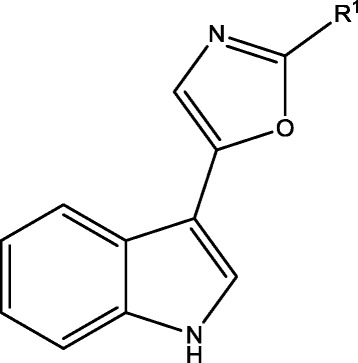
**Chemical structures of pimprinine and its analogues.** Pimprinine: R^1^ = CH3; Pimprinethine: R^1^ = CH2CH3; WS-30581 A: R^1^ = CH2CH2CH3; WS-30581 B: R^1^ = CH2CH2CH2CH3.

**Figure 2:**
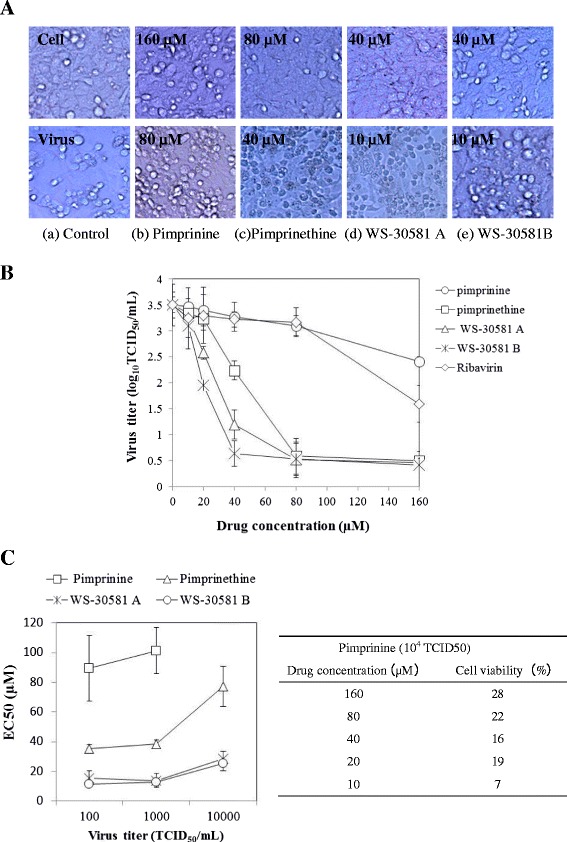
**Antiviral activities of pimprinine, pimprinethine, WS-30581 A and WS-30581 B against EV71 in a dose-dependent manner in RD cells. (A)** The inhibition of virus-induced CPE. RD cells were infected with 100 TCID_50_ of EV71 mixed with serial dilutions of tested compounds for 1 h at 37°C, the inocula were aspirated and the cells were incubated with DMEM/tested compounds at 37°C, 5% CO_2_ for 48 h, the antiviral effects were observed in terms of cellular morphology. **(B)** The inhibition of progeny virus yield. RD cells were infected with 100 TCID_50_ of EV71 in the absence or presence of tested compounds, the culture media and cell lysates were collected for virus titration at 10 h pi. The viral titers were presented as Log10 TCID_50_/mL. **(C)** Analysis of the dependence of the EC50s on virus titers. RD cells were infected with various TCID_50_ of EV71, the tested compounds were added as described in **(A)**, cell viability was determined at 48 h pi and the EC50 were calculated. The x-axis is in a base 10 logarithmic scale. Each value is the mean of triplicate assays ± standard deviation (SD).

The antiviral activity of pimprinine and its analogues pimprinethine, WS-30581 A and WS-30581 B (Figure 1), against EV71 based on inhibition of virus-induced cytopathic effects (CPE) in human rhabdomyosarcoma (RD) cells was examined. The cytotoxic effects were also evaluated. Table 1 shows that the effective concentrations 50% (EC50s) of pimprinine, pimprinethine, WS-30581 A and WS-30581 B were 89 μM, 35 μM, 16 μM and 11 μM against EV71, respectively and the selectivity indexes (SI) were 16, 24, 23 and 21, respectively. These compounds showed more potent inhibitory activity against EV71 than ribavirin (EC50 = 102 μM, SI = 12). As shown in Figure 2A, the EV71-infected cells showed a rounded-up appearance and detached from the dish in the absence of tested compounds. Treatments of RD cells with pimprinine, pimprinethine, WS-30581 A and WS-30581 B produced a slight protection against EV71-induced CPE at the lower concentrations, while a nearly complete inhibition of EV71-induced CPE was observed at the higher concentrations, which was used for the subsequent experiments in this study.

**Table 1:** **Antiviral activity against EV71, cytotoxicity, and selectivity index (SI) of pimprinine, pimprinethine, WS-30581 A and WS-30581 B in RD cells**

**Tested compound**	**CC50** ^**a**^ **(μM)**	**EC50** ^**b**^ **(μM)**	**EC90** ^**c**^ **(μM)**	**SI** ^**d**^
Pimprinine	1455 ± 27^e^	89 ± 18	149 ± 12	16
Pimprinethine	835 ± 20	35 ± 4	42 ± 3	24
WS-30581 A	363 ± 13	16 ± 1	26 ± 6	23
WS-30581 B	229 ± 10	11 ± 3	18 ± 2	21
Ribavirine^f^	1230 ± 355	102 ± 68	136 ± 49	12

^a^CC50, compound concentration required to reduce cell viability by 50%.

^b^EC50, compound concentration required to achieve 50% protection from virus-induced cytopathogenicity.

^c^EC90, compound concentration required to achieve 90% virus yield reduction.

^d^SI (selectivity index), ratio CC50/EC50.

^e^Values represent the mean ± SD of three independent experiments.

^f^Ribavirin, used as a positive control.

The protective effects of pimprinine, pimprinethine, WS-30581 A and WS-30581 B on EV71-induced CPE formation were confirmed by quantifying the effects on progeny viral yield. To this end, the confluent monolayers of RD cells in a 96-well plate were infected with 100 TCID_50_ of EV71 mixed with or without the tested compounds at various concentrations. After 10 h, the culture media and cell lysates were collected following freeze-thaw cycles and then subjected to virus titration. Treatments with tested compounds resulted in efficient and concentration-dependent reductions in progeny virus titers (Figure 2B), with a reduction of approximately 0.5 log for pimprinine and a 3.0 log reduction for pimprinethine, WS-30581 A and WS-30581 B relative to virus control group at a concentration of 80 μM. As a positive control, there was an approximate 2.0 log reduction in progeny virus titers with ribavirin at a concentration of 160 μM. The EC90 values derived from progeny viral yield assays are presented in Table 1.

To test the dependence of the EC50 values on virus concentrations, the RD cells were infected with various TCID_50_ of EV71 in the presence of a series dilutions of tested compounds for 1 h at 37°C. Inocula were removed and the cells were incubated further with various concentrations of the tested compounds in Dulbecco’s modified Eagle’s medium (DMEM) for 48 h. Antiviral activities were evaluated by determining cell viability with an 3-(4, 5-dimethylthiazol-2-yl)-2, 5- diphenyltetrazo-liumbromide (MTT)-method and the EC50s were calculated. As seen in Figure 2C, the EC50 values for pimprinethine, WS-30581 A and WS-30581 B were similar, with slight variations independently of the virus inoculum. Inhibition was eliminated when the virus concentration increased to above 10^4^ TCID_50_. For pimprinine, the anti-EV71 activity appeared to be largely dependent of the virus inoculum, and there was no inhibition observed when the concentration of virus increased to over 10^4^ TCID_50_. These results suggest that these compounds can be overwhelmed by excessive amounts of virus.

### Preliminary studies on the mechanisms of action of pimprinethine, WS-30581 A and WS-30581 B

Due to the lesser antiviral efficacy of pimprinine compared with the other compounds tested in our assay, pimprinethine, WS-30581 A and WS-3058 B were selected to further investigate the mechanisms of the antiviral action.

To determine if the pimprinine family of compounds inactivated virions directly, 10^3^ TCID_50_ of EV71 suspension was incubated in the presence of 80 μM pimprinethine, 40 μM WS-30581 A or 40 μM WS-30581 B for 24 h at 4°C. Subsequently, the viral titers in the mixture were measured by inoculating 10-fold dilutions of the mixtures beyond the effective concentrations of the compounds into the host cells. The TCID_50_ were calculated by the Reed and Muench method [21] on day 2 post inoculation; 160 μM ribavirin was used as a positive control. No significant difference was found between virus titers of the mixture for EV71 with and without the tested compounds present (data not shown). This evidence suggests that all compounds are not virucidal with respect to the viruses tested.

**Figure 3:**
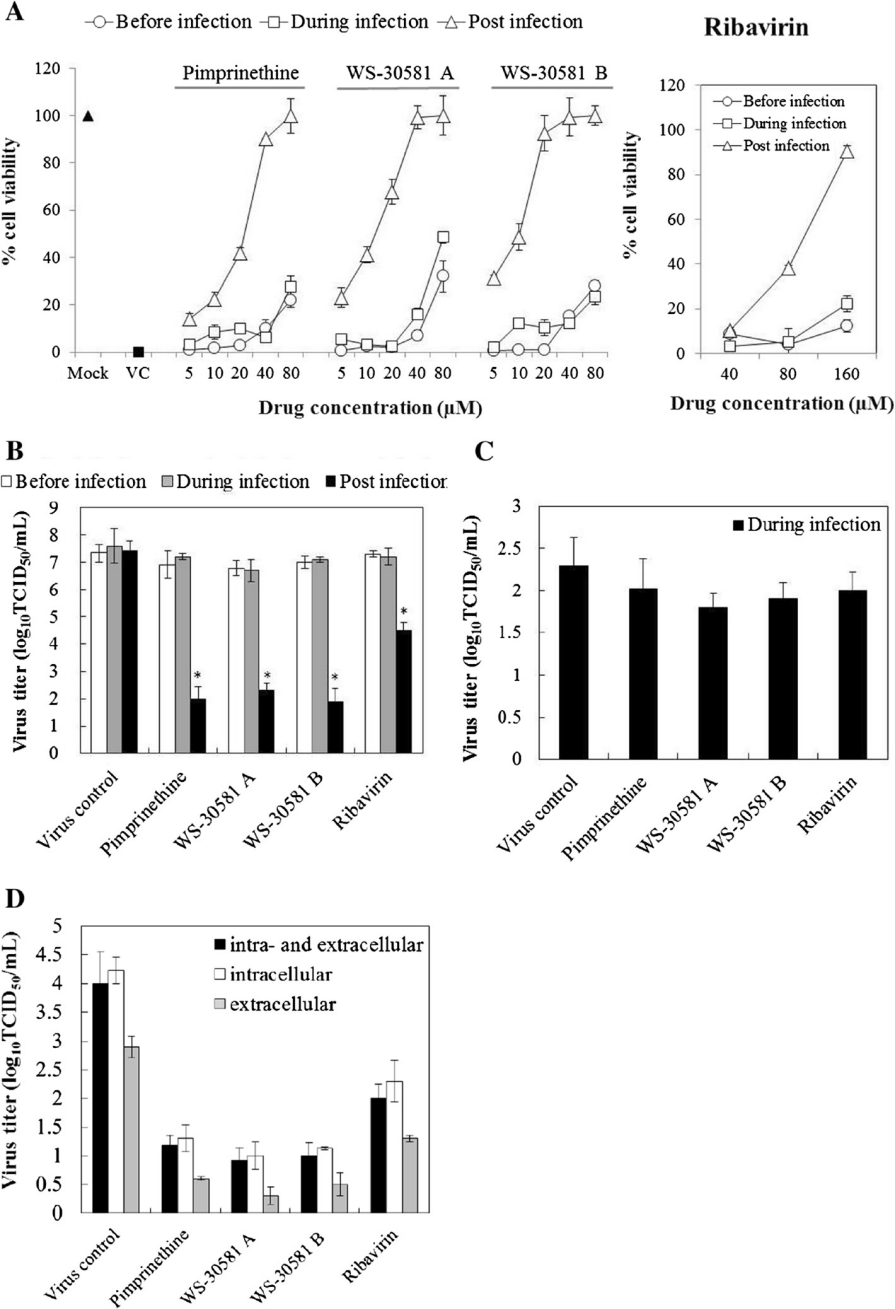
**Analysis of the modes of action of pimprinethine, WS-30581 A and WS-30581 against EV71. (A-B)** Analysis of the effective stage. The RD cells were incubated under serial 2-fold dilutions of tested compounds before, simultaneously or after EV71 (100 TCID_50_) inoculation. Antiviral effects were detected by measuring cell viability (A) and the progeny virus yields (80 μM) (B) after 48 h of infection. **(C)** Analysis of the effects on EV71 adsorption. Mock- or 160 μM pimprinethine, 80 μM WS-30581A and 80 μM WS-30581 B-treated EV71 (10^4^TCID50) was inoculated onto RD cells and adsorbed for 2 h, the infected cells were harvested and then subjected to virus titrations using the TCID_50_ method. **(D)** The effects on EV71 release from RD cells. RD cells infected with 100 TCID_50_ of EV71 were incubated with tested compounds for 12 h, both of cells and supernatants (intra- and extracellular), or one of which was harvested separately for determination of virus yield. Mock: no infection; VC, virus control. Values represent the means ± SDs of three independent experiments. *P <0.05, compared with virus control group.

To identify the stage in the viral life cycle that is affected by the pimprinethine, WS-30581 A and WS-30581 B, the assays were performed using three different treatment protocols. As shown in Figure 3A and B, all of the tested compounds exhibited the most powerful therapeutic effects that the viability rates of the infected cells treated with pimprinethine, WS-30581 A and WS-30581 B at concentrations of 80 μM were almost 100%. The virus titers of compounds-treated cells were much lower (approximately 5.0 log reduction for the three compounds) than those from untreated cells. On the contrary, a gradual loss in the antiviral effects was observed when the drugs were added just before or during infection. Ribavirin showed the same results as the tested compounds. These results suggest that pimprinethine, WS-30581 A and WS-30581 B are not preventive against EV71, neither do they inhibit adsorption of EV71, they mainly block the post-attachment stage of a viral infection.

This conclusion on adsorption inhibition was confirmed by intracellular virus titrations using the TCID_50_ method. As can be seen in Figure 3C, no significant decrease in the virus titer during virus attachment in the presence of either pimprinethine, WS-30581 A, WS-30581 B or ribavirin was detected, confirming that virus adsorption is not quantitatively affected by any of these compounds.

The possibility was tested that viral release from cells is affected by pimprinethine, WS-30581 A and WS-30581 B. Ribavirin was, once again, used as a positive control. Figure 3D shows that the virus titers from infected RD cells, the supernatants or total solutions (cell lysis solutions and the supernatants) treated with tested compounds have been significantly reduced, and a stronger inhibition of virus titers from the infected RD cells than those from the supernatants was observed, implying that the release of EV71 had been unaffected.

### Pimprinethine, WS-30581 A and WS-30581 B affect viral early steps of replication in cells

**Figure 4:**
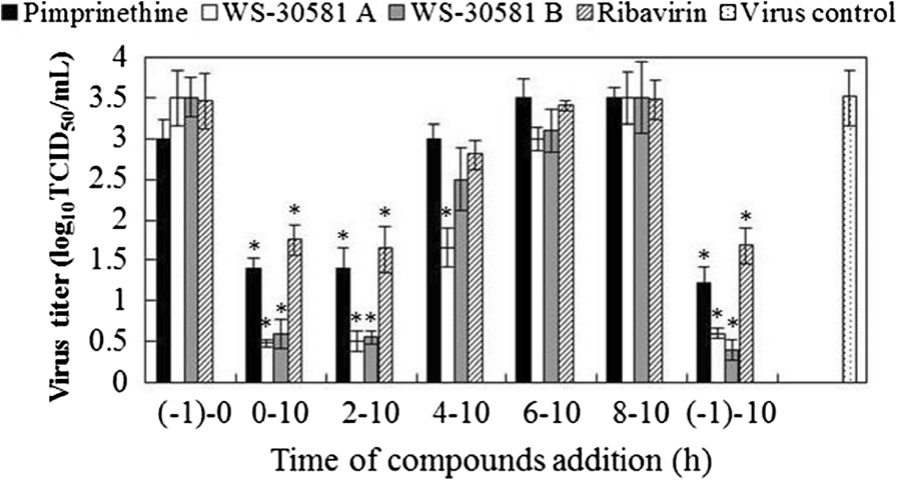
**Time-of-addition assay.** 80 μM pimprinethine, 40 μM WS-30581 A or 40 μM WS-30581 B were added to RD cells at different time periods after EV71 infection. At 12 h pi, the progeny virus yield was determined (-1–0 h: viral infection period; 0–10 h: the period for virus proliferation in the cells). Values are represented as the means ± SDs. *P <0.05, compared with virus control group.

In order to further understand the mechanisms of pimprinethine, WS-30581 A and WS-30581 B action against EV71 propagation in cells, a time-of-addition experiment was performed. As shown in Figure 4, when the tested compounds were present for the whole course of the replication cycle (-1–10), the titers of the progeny virus were reduced, which was similar to that for the addition of drugs during the 0–10 h and 2–10 h stage after viral infection (pi). For drugs treatments during other periods following EV71 infection, a gradual increase in viral yields was observed, reflecting a loss of the antiviral effects of the compounds. The control compound, ribavirin, exhibited a very similar trend. Results from this experiment indicate that these compounds act mainly at the early stage of the viral replication post infection.

### Pimprinethine, WS-30581 A and WS-30581 B inhibit strongly viral replication in RD cells

To investigate the effects of pimprinethine, WS-30581 A and WS-30581 B on EV71 replication further, the efficacy of the compounds in inhibiting progeny viral yields, viral RNA synthesis, and translation of viral protein were analyzed. Infected cells treated with or without 80 μM pimprinethine, 40 μM WS-30581 A or 40 μM WS-30581 B were harvested after 4, 8, 24 and 36 h p.i. in order to determine the progeny viral yields using the Reed and Muench method. Quantitative reverse transcription polymerase chain reaction (RT-PCR) and indirect immunofluorescence analysis of harvested cells were also carried out to determine the relative amounts of viral RNA and viral protein, respectively.

**Figure 5:**
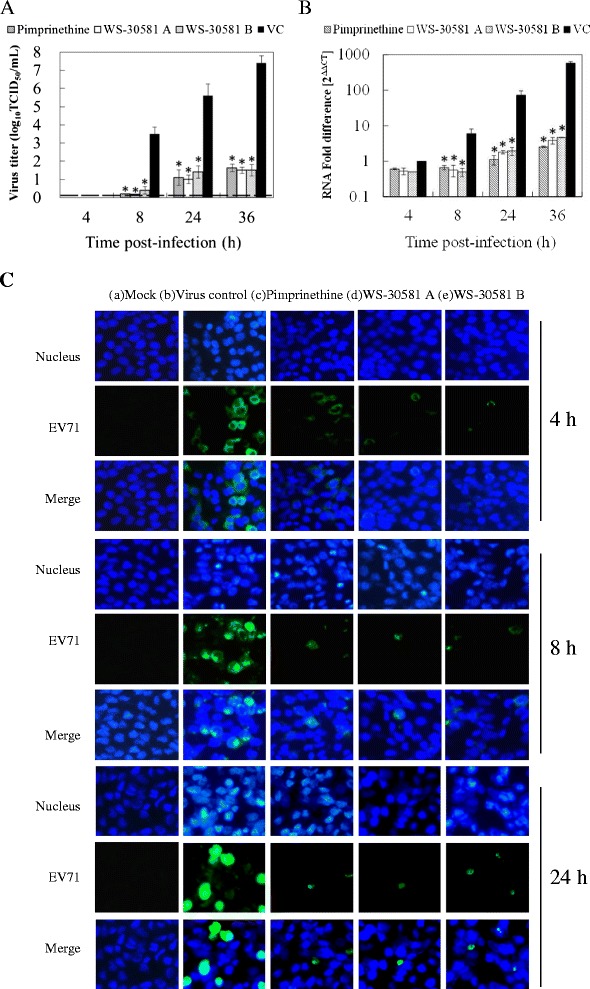
**Effects of pimprinethine, WS-30581 A and WS-30581 B on EV71 replication in RD cells.** RD cells infected with 100 TCID_50_ of EV71 were incubated in the absence (VC) or presence of 80 μM pimprinethine, 40 μM WS-30581 A or 40 μM WS-30581 B and harvested at the indicated times pi. **(A)** The progeny viral yields were determined. Dashed lines indicate virus titer less than the detectable dose. **(B)** The total RNA was extracted from cells and culture supernatants and EV71 RNA levels were measured. Cellular actin amplification was used for normalization. The △△CT data were calculated from three independent experiments. *P <0.05, compared with the virus control group. **(C)** EV71-protein was determined by indirect immunofluorescence using a mouse anti-enterovirus 71 monoclonal antibody and an Alexa Fluor 488-Conjugated Affinipure Goat Anti-Mouse IgG (H + L). The nucleus was stained with DAPI and the green foci indicate the presence of EV71 protein.

As shown in Figure 5A and B, the virus titer and the level of viral RNA continued to increase from 4 to 36 h in the virus control cells, which indicated the virus was actively replicating in the cells following the viral inoculation. Notably, no obvious change obviously was observed in the cells treated with compounds, and inhibitory effects were the most prominent at 36 h p.i. This result implies that pimprinethine, WS-30581 A and WS-30581 B target viral replication in RD cells.

The influence of the tested compounds on EV71 replication at the level of translation was also determined. Immunofluorescence foci of viral protein were not observed in the mock-infected control (Figure 5C-a), which suggested that the antibody was specific for EV71. The green immunofluorescence foci in the virus control group (Figure 5C-b) were significantly more abundant than that in the compounds-treated cells (Figure 5C-c, d, e), indicating that the viral protein synthesis was suppressed by the compounds as a result of their cumulative inhibitory action on viral RNA synthesis. These results lead us to propose that pimprinethine, WS-30581 A and WS-30581 B suppress EV71 replication by inhibiting viral RNA and protein synthesis.

### Pimprinethine, WS-30581 A and WS-30581 B inhibit EV71-induced apoptosis

**Figure 6:**
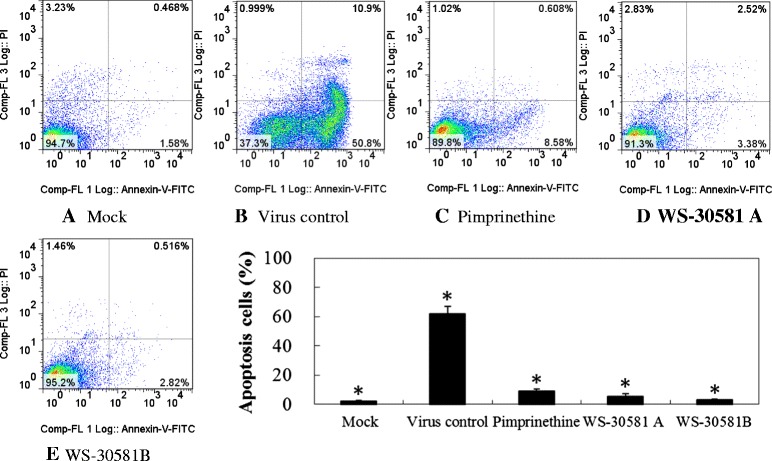
**The inhibitory effects of pimprinethine, WS-30581 A and WS-30581 B on EV71-induced apoptosis.** RD cells were left untreated **(A)** or were infected with 100 TCID50 of EV71, after viral adsorption, RD cells were incubated in the absence **(B)** or presence of 80 μM pimprinethine **(C)**, 40 μM WS-30581 A **(D)** and 40 μM WS-30581 B **(E)** for 36–48 h, the cells were stained with Annexin-V- fluorescein and propidium iodide and measured using flow cytometry. Values are represented as means ± SDs. *P <0.05, compared with virus control group.

Previous studies have shown that EV71 induces apoptosis in infected cells when viral protein synthesis occurs, whereas neither viral adsorption, internalization, entry, uncoating, nor viral RNA replication are required to trigger this apoptosis [22]. Flow cytometry was performed to investigate the effects of pimprinethine, WS-30581 A and WS-30581 B on virus-induced cell apoptosis. As illustrated in Figure 6, RD cells infected with EV71 (virus control) showed a significant fluorescence drift to the right (representative of early apoptosis) and to the upper-right quadrant (representative of late apoptosis or death) in comparison to the mock-infected cells. While fluorescence drifting could be hardly observed with addition of pimprinethine, WS-30581 A and WS-30581 B. These data demonstrate that these compounds can effectively inhibit the EV71-induced apoptosis in RD cells, which indirectly reflects their inhibition of viral protein synthesis.

### Antiviral activities of pimprinine family compounds towards other human viruses

The antiviral efficacies of pimprinine, pimprinethine, WS-30581 A and WS-30581 B against representatives of several classes of virus were also evaluated in cell-based assays. The viruses used for testing included: non-enveloped RNA virus, CVB3; enveloped RNA virus, influenza virus A/human/Hubei/86/2009 (H1N1); enveloped DNA virus, HSV-1 and non-enveloped DNA virus, ADV-7. Ribavirin, amantadine and acyclovir were used as reference inhibitors. Cytotoxicity on Hep-2, HeLa and MDCK, human cell lines suitable for the replication of these viruses respectively, were also evaluated using an MTT method.

**Figure 7:**
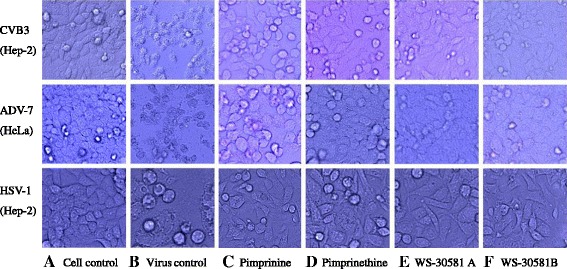
**Antiviral activity of pimprinine, pimprinethine, WS-30581 A and WS-30581 B against CVB3, ADV-7 and HSV-1.** The Hep-2 and HeLa cells were left untreated **(A)** or infected with 100 TCID50 of CVB3, ADV-7 and HSV-1, after virus inoculation, the infected cells were mixed without **(B)** or with 160 μM pimprinine **(C)**, 80 μM pimprinethine **(D)**, 40 μM WS-30581 A **(E)** or 40 μM WS-30581 B **(F)** for 1 h at 37°C respectively, the inocula were aspirated and the cells were further incubated with DMEM/tested compounds at 37°C, 5% CO_2_ for 48 h, the antiviral effects were observed with respect to cellular morphology.

Since CVB3, ADV-7 and HSV-1 infections caused marked CPE, we started by evaluating the antiviral properties of these compounds through the analysis of CPE formation in CVB3-infected Hep-2 cells, ADV-7-infected HeLa cells and HSV-1-infected Hep-2 cells. Figure 7 shows that these compounds could protect cells from virus-induced CPE. Morphologically, the cells infected with viruses, in the absence of tested compounds, showed typical CPE such as the rounding and formation of giant multinucleated syncytia (Figure 7-B) compared with mock infection (Figure 7-A). CPEs of infected cells were inhibited by treating them with 160 μM pimprinine (Figure 7-C), 80 μM pimprinethine (Figure 7-D), 40 μM WS-30581 A (Figure 7-E) or 40 μM WS-30581 B (Figure 7-F).

These compounds exhibited high to moderate activity against the different viruses, the rank order of virus sensitivity was ADV-7 > CVB3 > HSV-1 > H1N1 (Table 2). All compounds were inhibitory against ADV-7 and CVB3, and WS-30581 A and WS-30581 B were approximately 7-fold more active than ribavirin (Table 2). Pimprinethine, WS-30581 A and WS-30581 B also exhibited significant antiviral activity against HSV-1, and were capable of achieving approximately 80% inhibition of HSV-1-induced plaque formation. Acyclovir exhibited strong inhibition of HSV-1 (Table 2). However, WS-30581 A and WS-30581 B produced less than 70% inhibition of H1N1 infection and pimprinine and pimprinethine demonstrated negligible measurable inhibition. Amantadine produced a good anti-H1N1 effect in this assay (Table 2).

**Table 2:** **Inhibitory effects of pimprinine, pimprinethine, WS-30581 A and WS-30581 B against other human viruses**

**Tested compound**		**CVB3**	**HSV-1**	**ADV-7**	**H1N1**
	CC50^a^	443 ± 35^e^	443 ± 35	1140 ± 181	899 ± 56
Pimprinine	EC50^b^	95 ± 16	160 ± 18	150 ± 10	—
	EC90^c^	—^f^	—	266 ± 14	—
	SI^d^	5	3	8	—
	CC50	256 ± 49	256 ± 49	750 ± 30	577 ± 73
Pimprinethine	EC50	22 ± 4	45 ± 3	33 ± 6	—
	EC90	58 ± 9	—	63 ± 5	—
	SI	12	6	23	—
	CC50	72 ± 8	72 ± 8	217 ± 4	212 ± 18
WS-30581 A	EC50	14 ± 4	18 ± 2	17 ± 18	38 ± 7
	EC90	39 ± 4	—	43 ± 3	—
	SI	5	4	13	6
	CC50	83 ± 6	83 ± 6	354 ± 14	215 ± 28
WS-30581 B	EC50	12 ± 2	14 ± 4	15 ± 2	24 ± 5
	EC90	42 ± 6	—	48 ± 6	—
	SI	7	6	24	9
	CC50	1311 ± 385		1516 ± 243	
Ribavirin^g^	EC50	90 ± 10		116 ± 19	
	EC90	103 ± 17		132 ± 22	
	SI	15		13	
	CC50		>2000		
Acyclovir^h^	EC50		11 ± 1		
	EC90		24 ± 5		
	SI		>181		
	CC50				>2000
Amantadine^i^	EC50				73 ± 8
	EC90				90 ± 12
	SI				>27

^a^CC50, compound concentration required to reduce cell viability by 50%.

^b^EC50, compound concentration required to achieve 50% protection from virus-induced cytopathogenicity.

^c^EC90, compound concentration required to inhibit 90% virus yield.

^d^SI (selectivity index), ratio CC50/EC50.

^e^Values represent the mean ± SD of three independent experiments.

^f^—, less than 50% or 90% inhibition; ^g, h, i^Ribavirin, acyclovir and amantadine, used as positive controls.

Virus yield reduction assays were also carried out to evaluate further the protective effects of the tested compounds. Pimprinine, pimprinethine, WS-30581 A and WS-30581 B showed activities that were similar to the CPE inhibition results. The dose-dependent effectiveness for antiviral activity, expressed as EC90s, is shown in Table 2.

### Pimprinethine, WS-30581 A and WS-30581 B also inhibit CVB3, ADV-7, HSV-1 and H1N1 replication in cells

To investigate which stage pimprinethine, WS-30581 A and WS-30581 B inhibit viruses infection at, various concentrations of these compounds were incubated with Hep-2 cells, HeLa cells and MDCK cells before (before infection), simultaneously (during infection) or after (post infection) CVB3, ADV-7 and H1N1 inoculation. CPEs were observed and the viability of the cells was determined with MTT assays after incubation at 37°C for 48 h. For HSV-1, the inhibitory effects were measured using plaque reduction assays on inoculated Hep-2 cells.

**Figure 8:**
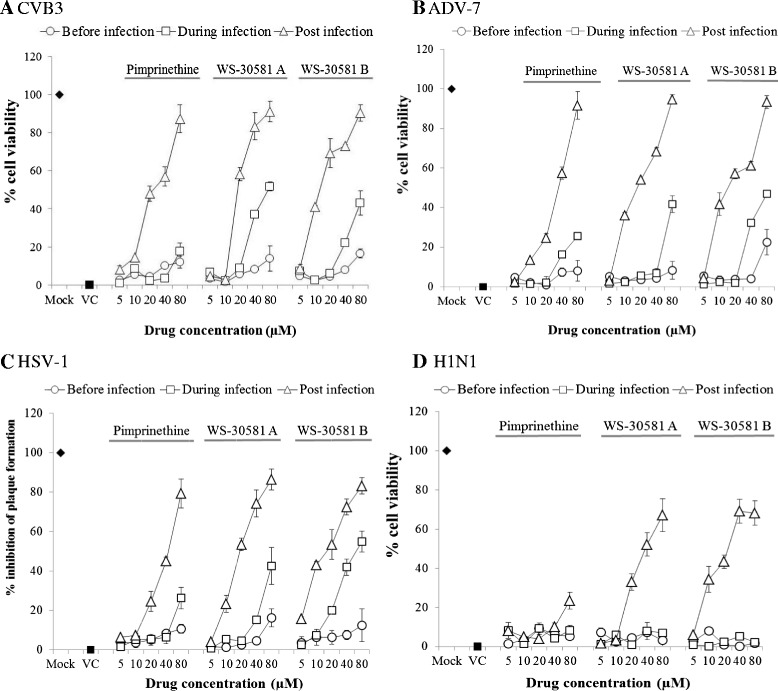
**Analysis of effective stages of pimprinethine, WS-30581 A and WS-30581 B against four viruses: CVB3, ADV-7, HSV-1 and H1N1.** The cells were infected with different viruses, and incubated under serial 2-fold dilutions of tested compounds at the indicated periods (before, simultaneously or after virus inoculation) following different procedures, the antiviral effects were detected by measuring of cell viability (**A**: CVB3, **B**: ADV-7 and **D**: H1N1) or plaque formation (**C**: HSV-1). Mock: no infection; VC, virus control. Values represent the means ± SDs of three independent experiments.

As shown in Figure 8, all compounds analyzed showed dose-dependent inhibitory effects on viral replication under the conditions of post-infection. Treatment with the compounds before infection or during virus infection produced little or no antiviral effect on any of the viruses, indicating that the antiviral target is the replication stage of the virus. This finding suggests that similar modes of inhibition operate in all of the viruses tested. These results are compatible with the conclusion drawn previously, which was that pimprinethine, WS-30581 A and WS-30581 B target the viral replication stage.

## Discussion

EV71 infections have received much attention because of the increased incidences and lack of vaccines and effective therapies. Natural substances offer an interesting pharmacological perspective for the development of novel antiviral drugs with broad-spectrum antiviral properties and novel modes of action. Some natural indole alkaloid compounds have been proven to have antiviral potential against dengue virus and HSV-2 [23,24]. In this study, we report novel findings that pimprinethine, WS-30581 A and WS-30581B, as natural indole alkaloids, were effective inhibitors of the replication of EV71 and ADV-7, with slight inhibitory activity against CVB3, HSV-1 and H1N1 (with a few exceptions). Pimprinine, however was only slightly inhibitory or inactive against all of these viruses.

The chemical structures of pimprinine, pimprinethine, WS-30581 A and WS-30581 B differ by a methyl group (Figure 1A), which must ultimately lead to the variations in antiviral activity and cytotoxicity. Thus, compounds with better antiviral efficacies could possibly be produced by modifying these structures, which could also be confirmed further by some of the synthetic pimprinethine analogues with various more excellent biological activities [25,26].

EC50 values derived from three-day assays are usually more predictive of in vivo efficacy than 48-hr assays. We also measured the CPEs of the cells infected with the five viruses after three days of incubation with tested compounds in 96-well plates, where the virus inoculum reached >90% CPE. The results were similar to those obtained from treatments with tested compounds for two days (data not shown). This also showed the effectiveness and stability of these compounds against viral infections, especially of EV71.

The SI of pimprinethine, WS-30581 A and WS-30581 B against EV71 were better than that of the control compound, ribavirin, in RD cells (Table 1). Although all tested compounds were more toxic than ribavirin, however they could inhibit the replication of EV71 at lower concentrations. This provides evidence that the compounds exhibit cytotoxic effects on the host cells after an antiviral role, rather than destroying cells directly and hence inhibiting viral proliferation within them. For compounds with lower SI values, it is difficult to refute the possibility that they may damage cells or slow down cell metabolism enough to inhibit virus production at or near their EC50. In our assays the EC50s of all compounds tested, against five human viruses, were measured by the observation of CPE microscopically and from the determination of cell viability with MTT assays and virus yield reduction assays. CPE can reflect the surface morphology of infected cells, while the MTT assay measures the level of cellular metabolism, and virus yield assays tests viral multiplication directly in infected cells. The similarity of the EC50s obtained for all compounds using the three different approaches suggests that the compounds alone do not inhibit cell growth. Furthermore, the successful use of pimprinine as a significant protection against electrically induced convulsions *in vivo* (80 mg/kg) [19] suggests that the toxicity observed in cell culture may not be equivalent to that *in vivo*. Previous research has demonstrated that the acute toxicity of WS-30581 A in ddY mice by intraperitoneal injection was above 250 mg/kg [18]. These findings suggest that the pimprinine family of compounds have potential therapeutic applications.

Since the CC50 values for pimprinine and pimprinethine have been found to be extremely high, the cytotoxic effects of all the tested compounds against RD cells, and pimprinine and pimprinethine against HeLa, Hep-2 and MDCK cells, have also been confirmed using an ATPLite luminescence-based assay (Perkin Elmer, Waltham, MA). The results were shown in Table 3. The ATPLite assay may be more sensitive to cytotoxicity than the MTT method. For RD and Hep-2 cells, similar results were obtained, and for HeLa and MDCK cells, the values of CC50 from the ATPLite assay were smaller than those from MTT assays (Tables 1, 2 and 3).

**Table 3:** **Cytotoxicity of pimprinine, pimprinethine, WS-30581 A and WS-30581 B in RD, Hep-2, HeLa and MDCK cells**

	**CC50** ^**a**^ **(μM)**			
**Tested compound**	**RD**	**Hep-2**	**HeLa**	**MDCK**
Pimprinine	1263 ± 247	403 ± 53	940 ± 219	649 ± 77
Pimprinethine	656 ± 82	233 ± 36	550 ± 103	406 ± 48
WS-30581 A	313 ± 49	—^b^	—	—
WS-30581 B	267 ± 31	—	—	—

^a^CC50, compound concentration required to reduce cell viability by 50%.

^b^—, not done.

The EV71 replication cycle can be divided into the following steps: viral attachment, entry, polyprotein translation and cleavage, viral RNA replication, assembly and release [27]. These critical steps are currently considered to be the targets for the development of antiviral agents [28]. Picornavirus could complete its life cycle in 5–10 h (approximately 8 h). Upon virus attachment and entry into the host cell, an uncapping event occurs to release the RNA genome into the cell. Cap-independent translation of the viral RNA takes place through the recruitment of host replication machinery. Negative RNA intermediates of the viral genome are also generated to serve as templates for the replication of positive-sense RNA viral genomes. These events have been predicted to reach high levels at 3–4 h post infection. Progeny virions are then self-assembled from the synthesized viral proteins and RNA genomes, which begins in the cytoplasm during the 4–6 h, and the release of virus particles is conducted during the 6–10 h period [29-31]. In our assays, time-addition assays with pimprinethine, WS-30581 A and WS-30581 B demonstrated that inhibition of virus yields declined when RD cells were treated with these compounds at more than 4 h post infection, which was consistent with EV71 genomes replication and protein synthesis being at a high level during 3–4 h post viral infection. These compounds showed strong activity against viral RNA synthesis, but not completely inhibited viral protein synthesis. These results allow us to conclude that there is a direct effect of the compounds on viral RNA synthesis, and the inhibition of viral protein synthesis by the compounds is the result of their cumulative inhibitory action on viral RNA synthesis. More detailed analyses of the mechanisms of action of these compounds are currently being conducted.

To date several mechanisms of ribavirin action have been proposed. These include: (a) Inhibition of inosine monophosphate dehydrogenase (IMPDH) [32]; (b) Inhibition of proinflammatory mediators induced by viral infection [33] and (c) Inducement of lethal mutagenesis after incorporation during viral RNA synthesis, which leads to a loss of total viral genomic RNA [34]. Pimprinethine, WS-30581 A and WS-30581 B had greater antiviral activity than ribavirin, but similar results were obtained for all of these compounds to determine the mechanisms of antiviral action. Therefore, it is conceivable that these antiviral agents act according to some, but not all, of the mechanisms of action that have been proposed for ribavirin.

Pimprinine is an MAO inhibitor, so it could be interesting to find out whether other MAO inhibitors (such as TCP and pargyline) also possess anti-enterovirus activity. We have tested whether TCP and pargyline have antiviral activity against EV71 and CVB3 and found them to be inactive. This suggests that the mode of antiviral action of these compounds is likely to differ to their mechanisms of anti- MAO action.

EV71 induced apoptosis has been considered to be an important mechanism in disease pathogenesis [35]. Apoptosis leads to the spread of viral progeny, which may cause viremia and severe central nervous system complications. In this study, pimprinethine, WS-30581 A and WS-30581 B were found to have an obvious inhibitory effect on EV71-induced apoptosis, which may have a significant impact on protecting hosts from the severe consequences of EV71 infection.

Pimprinethine, WS-30581 A and WS-30581 B have shown post-exposure activity on EV71-infected cells. We also demonstrated that these compounds target the post-infection stages of CVB3, ADV-7, HSV-1 and H1N1 life cycles. Thus, the pimprinine family of compounds may be safe and effective agents for therapeutic use against EV71 infection, and therapeutic agents for the adjuvant treatment of other viral infections.

## Conclusions

Pimprinethine, WS-30581 A and WS-30581 B exhibited inhibitory activity against EV71 and ADV-7, slightly activity against CVB3, HSV-1 and H1N1, with a few exceptions, *in vitro*. These compounds mainly act at the early stage of the EV71 replication period. The mechanism by which these antiviral agents act against other viral infections may be similar to that shown for EV71. The data described herein demonstrate that the pimprinine family of compounds are effective therapeutic agents for the treatment of EV71 infection. Here we have uncovered new information about the scope of the biological activity of the pimprinine family of compounds.

## Materials and methods

### Cells, viruses and tested compounds

Human rhabdomyosarcoma cells (RD), Human laryngeal carcinoma cells (Hep-2), Human cervical carcinoma cells (HeLa) and Madin-Darby canine kidney cells(MDCK)(purchased from China Center for type Culture Collection, CCTCC) were maintained in DMEM (Gibco) supplemented with 10% fetal bovine serum (FBS; Gibco), 100 U/mL of penicillin and streptomycin, and 2 mM L-glutamine. EV71 (XiangYang-Hubei-09), CVB3 (Nancy strain), ADV-7, HSV-1 and H1N1 were kind gifts from Professor Zhanqiu Yang (Institute of Medical Virology, School of Medicine, Wuhan University, China) and were propagated in RD, Hep-2, HeLa and MDCK cell lines. Viral titers were determined using the standard method of median tissue culture infective dose (TCID_50_) on corresponding host cells [21]. Pimprinine, pimprinethine, WS-30581 A and WS-30581 B (Figure 1A) were isolated from Streptomyces sp. WS-13317 and identified by comparing spectra to those in the literature [18,36]. Amantadine, ribavirin and acyclovir used as positive controls, were purchased from Sigma Chemical Co. Stock solutions of drugs were prepared in dimethyl sulfoxide (DMSO) at a final concentration of 0.1% and diluted with maintenance medium (MM) consisting of DMEM with 2% fetal bovine serum.

### Determination of cell viability

Cell viability was assessed by an MTT assay, which functions based on the reduction of a MTT into formazan dye by active mitochondria. The cells were treated with 100 μL of MTT (1 mg/mL, Sigma) and incubated at 37°C for 4 h. The reaction was blocked by DMSO and measured in a microplate reader (Bio-Tek Instruments) at 492 nm. The untreated control was arbitrarily set as 100%.

### Plaque reduction assay

Confluent monolayers of Hep-2 cells, seeded in a 24-well plate, were infected with HSV-1 at a density of 60–80 plaque-forming units (PFU) per well and treated with media (pre-warmed DMEM containing 2% FBS, 0.8% low-melting agarose) mixed with or without the serially diluted tested compounds for the periods indicated following different procedures. The cells were incubated at 37°C until plaques appeared, followed by fixing with 10% formaldehyde and staining with 0.5% crystal violet. The plaques were counted by visual examination. The ratio of the number of plaques in the treated group to that in the untreated control was calculated.

### Antiviral activity and cytotoxicity

The antiviral activities of pimprinine, pimprinethine, WS-30581 A and WS-30581 B against EV71, ADV-7, CVB3, HSV-1 and H1N1 were determined according to inhibition of virus-induced cytopathogenicity effects (CPE) in acutely infected RD, HeLa, Hep-2 and MDCK cells. Confluent cell monolayers in 96-well dishes were infected with 100 TCID_50_ of corresponding virus dilution mixed with serial dilutions of tested compounds for 1 h at 37°C. Inocula were aspirated and the cells were then incubated with various concentrations of DMEM/tested compounds at 37°C, 5% CO_2_ for 48 h. CPE were observed microscopically and the viability of the cells was determined using MTT assays. A plaque reduction assay was performed to measure the inhibitory effects of the test compounds against HSV-1 infection. The concentrations required for the tested compounds to achieve 50% protection of cells from virus-induced CPE were determined.

Adverse effects of pimprinine, pimprinethine, WS-30581 A and WS-30581 B on the host cells (RD, HeLa, Hep-2 and MDCK) were also assessed by means of the MTT-method, by exposing uninfected cells to various concentrations of tested compounds for 48 h at 37°C, the viability of the cells was subsequently determined. The 50% cell cytotoxic concentrations (CC50s) of compounds were calculated using SPSS software. SI were calculated from the ratio of CC50: EC50.

### Virus yield reduction assay

The virus suspension, serially diluted 10-fold with DMEM containing 2% FBS, was inoculated to cells in a 96-well plate. After 1 h incubation at 37°C in 5% CO_2_, unbinding virus was washed out and DMEM maintenance medium supplemented with 2% FBS was added to the cells. After 2 days, the infected cells were monitored for cytopathic effects (CPEs). The virus titer was calculated by the Reed–Muench method [21].

### Effective stage analysis

To identify the step in the viral life cycle that is affected by pimprinethine, WS-30581 A and WS-30581 B, various concentrations of tested compounds were added to cells according to the following three different treatment procedures. (i) To analyze for a preventive effect (before infection), the compounds were added to cells for 2 h at 37°C followed by washing with maintenance medium (MM) before virus infection. (ii) To analyze for inhibition of adsorption (during infection), the mixtures of the compounds and virus were added to cells for 2 h at 37°C followed by washing with MM. (iii) To analyze for a therapeutic effect (post infection), the cells were first infected for 1 h at 37°C followed by washing with MM, the compounds were added and incubated with the cells for the duration of the experiment. For all treatments, cell viability and progeny virus yields were measured after 48 h of infection.

### Adsorption analysis

Cells were infected with EV71 (10^4^ TCID_50_) containing 160 μM pimprinethine, 80 μM WS-30581 A or 80 μM WS-30581B, after 2 h adsorption at 37°C, inoculum was discarded, cells were washed three times with phosphate buffered saline (PBS) and harvested following freeze-thaw cycles, and subjected to virus titration. Cells infected with EV71, but treated in the absence of tested compounds, were used as a virus control.

### Release analysis

RD cells were treated with or without 80 μM pimprinethine, 40 μM WS-30581 A or 40 μM WS-30581 B after 100 TCID_50_ of EV71 infection; Supernatants and cells were harvested together or separately for determination of progeny virus yields at 12 h p.i. by the method of Reed and Muench [21].

### Time of (drug) addition experiment

RD cells were infected with 100 TCID_50_ of EV71 and then 80 μM pimprinethine, 40 μM WS-30581 A and WS-30581 B were added at different time phases (−1–0 h, 0–10 h, 2–10 h, 4–10 h, 6–10 h, 8–10 h and −1–10 h pi, where −1–0 h is the viral infection period and 0–10 h is the period for virus proliferation in the cells). The cells and supernatants were harvested at 10 h post-infection (pi) and subjected to three freeze-thaw cycles, after which the viruses were titered by the Reed–Muench method [21].

### RNA extraction and quantitative reverse transcription-PCR

The EV71 RNA was extracted from infected cells and culture supernatants with TRIzol (Invitrogen) and reverse-transcribed using a PrimeScript RT reagent Kit (Takara) according to the manufacturer’s instructions. The products of reverse transcription were quantified with the SYBR Premix Ex Taq II(perfect real time)Kit (TaKaRa) and detected with a Step One Plus sequence detection system (Applied Biosystems). Expression of actin was used as an internal standard. The special primer sequences were: EV71-VP1-F: 5'-CACACAGGTGAGCAGTCATCG-3', EV71-VP1-R: 5'-GTCTCAATCATGCTCTCGT CACT-3'; Actin-F: 5'-GGCGGGACCACCATGTACCCT-3', Actin-R: 5'-AGGGGCCGGA CTCGT CATACT-3'.

### Immunofluorescence assay

The RD cells infected with EV71 in a 24-well plate were fixed with 4% paraformaldehyde for 20 min and permeabilized with 0.5% Triton X-100 (in PBS) for 20 min, then blocked with 1% bovine serum albumin for 30 min, and subsequently incubated with the primary antibody (mouse anti-enterovirus 71 monoclonal antibody) for 2 h, followed by the appropriate Alexa-Fluor-488-labeled secondary antibody (Alexa-Fluor- 488-conjugated AffiniPure goat anti-mouse IgG (H + L)) for 60 min. Cell nuclei were stained with 40, 6-diamidino-2-phenylindole (DAPI). After each step, the slides were washed repeatedly with PBS with Tween (PBST). Fluorescence was observed and recorded using a confocal laser- scanning microscope.

### Flow cytometry analysis

For the apoptosis assay, the RD cells in 6-well plates infected with 100 TCID_50_ of EV71 were left untreated or treated with 80 μM pimprinethine, 40 μM WS-30581 A and WS-30581 B for 36–48 h, until the CPEs of virus control cells reached 70–80%. The cells were stained with Annexin-V-fluorescein and propidium iodide, according to the manufacturer’s instructions and subsequently subjected to flow cytometry analysis.

### Statistical analysis

Experimental results are expressed as means of at least three independent experiments. Values are expressed as means ± standard deviations (SD). Comparisons between experimental and control groups were performed using the unpaired Student’s t-test. In all cases, p <0.05 was considered significant.

## Abbreviations

CPE: Cytopathic effects
EC50: 50% effective concentration
CC50: 50% cytotoxic concentration
SI: Selectivity index
MTT: 3-(4,5-dimethylthiazol-2-yl)-2,5-diphenyltetrazolium bromide
DMSO: Dimethyl sulfoxide
PFU: Plaque-forming units
EV71: Enterovirus 71
CVB3: Coxsackievirus B3
ADV-7: Adenovirus type 7
HSV-1: Herpes simplex virus 1
